# Editorial: Psychocardiology then and now – the genesis of a discipline

**DOI:** 10.3389/fpsyt.2022.988393

**Published:** 2022-08-16

**Authors:** Don Byrne, Kai G. Kahl, Marlies E. Alvarenga

**Affiliations:** ^1^College of Health and Medicine, The Australian National University, Canberra, ACT, Australia; ^2^Department for Psychiatry, Social Psychiatry and Psychotherapy, Hannover Medical School, Hannover, Germany; ^3^Department of Psychology, Institute of Health and Wellbeing, Federation University Australia, Melbourne, VIC, Australia

**Keywords:** psychocardiology, serious mental disorder, allostasis, childhood adversity, psycho-biological interaction

The poet Schiller, reflecting on the happiest of emotions, says:

*The golden time of first love!*

*The eye sees the open heaven*,

*The heart is intoxicated with bliss;*

[from The Song of the Bell (Das Lied von der Glocke), 1798]

And from a rather more melancholic disposition, the poet Keats writes:

*My heart aches, and a drowsy numbness pains*

*My sense, as though of hemlock I had drunk …*

(from Ode to a Nightingale, 1819).

What then connects these exquisite lines? It is that the seat of such contrasting emotions has been placed explicitly in the human heart. Indeed, the range of emotions to which this attribution applies extends wider than these two poetic examples portray. Countless recorded works of poetry and prose, philosophy, and religion have held this view to be self-evidently true. And the compelling nature of the link remains with us now. But from the perspectives of both the psychological and cardiological sciences in the twenty-first century it is a conspicuous misattribution. It is, however, not difficult to understand how it could have occurred.

When we experience and identify an emotion, whether positive or negative, we are also reporting a set of explainable and measurable physiological events having their origins in the activation of key centers within the nervous system (1). The pioneering psychologist James (2) held that a consciously expressed emotion is little more than the subjective self-report of bodily changes arising from autonomic activation. And though challenged by Cannon (3) on neurophysiological grounds, this conceptualization prevailed for many decades as the dominant theoretical foundation for understanding and explaining human emotion.

The cardiovascular system has figured strongly in this causal reasoning. Activation of the autonomic nervous system can be subjectively experienced in a number of recognizable and disturbing cardiovascular events—rapid and irregular pulse, blushing and elevations of skin temperature, shortness or rapidity of breath, light-headedness, and syncope, all present themselves for consideration, but hemodynamic events reflecting fluid pressure in the circulatory system also enter this mix. The evidence for this assertion is now abundant and indisputable (4–6). But while the poet considers this link from an experiential viewpoint and concludes that the actual source of the feeling lies in the heart, the scientist, considering the same psycho-biological link, can now state with certainty, that while the heart reacts, the origins of the feeling lie clearly within the nervous system. So, for the purposes of the present discourse, while the heart is not the source of the feeling, it is undeniably a powerful barometer of that feeling.

The use of the term *psychocardiology* is relatively new (7, 8). But recognition of the role of psychological factors and risk or incidence of cardiovascular disease (CVD), goes much further back in history. A full analysis of the rise of psychocardiology over the past century is not intended of this Editorial. The sheer volume of contemporary literature documenting the heart/brain nexus is now formidable. Still, brief reference to some important milestones along the way will serve as a context for the presentation of the contemporary papers which will make up the substance of this Frontiers Special Issue exploring the heart/brain interface.

Passing over purely speculative references to the possibility of a heart/brain nexus which have appeared throughout medical history, the late nineteenth and early twentieth centuries began to see the more formal chronicling of that nexus based on systematic clinical observations. The extreme mental stress of military combat—the anticipation of a violent death in the immediate future—and its pathological impact on the heart, was first formally noted by Jacob Da Costa (9). The term *Soldier's Heart*, ultimately known as Da Costa's Syndrome, included palpitation, fatigue, breathlessness, chest pain, sighing, dizziness, and faintness (10). This constellation of physical symptomatology, at least contemporaneous with severe mental stress, is clearly indicative of significant cardiac distress, though there is no evidence either that it was protracted or that it gave rise to an elevated risk of clinically documented, acute cardiac events such as myocardial infarction. Nonetheless, clinical observation by military medical practitioners of the day did foreshadow, more than a century and a half ago, the possibility that the mind might seek expression through the cardiovascular system.

Looking to the general population more broadly, the pioneering Canadian physician William Osler (11) first noted, on the basis of clinical observation again, that a particular pattern of pressured and achievement driven behavior appeared to be conspicuously over-represented in patients presenting clinically with angina pectoris—anticipating perhaps, as we will see a little later, the formulation of the Type A behavior pattern. A short time later, the emergence of psychoanalytic theory and practice in the early twentieth century came to provide a more grounded basis for understanding both mental and somatic phenomena through carefully systematized clinical observation, giving rise to the now recognized medical discipline of *psychosomatic medicine* (12). And within the specific domain of cardiovascular disease, the influential psychoanalyst Franz Alexander reported that hypertensive patients tended to be those suppressing disturbing and problematic experiences deeply into the subconscious without being able to externally recognize, accept, and resolve these experiences (13).

The heart/brain nexus became subjected to the methodological and statistical rigors of cardiovascular epidemiology several decades later with the landmark work of the cardiologists Rosenman and Friedman. Evidence from the now iconic Western Collaborative Group Study (WCGS) (14) demonstrated prospectively, that individual possession of the Type A behavior pattern—interestingly similar to Osler's earlier observations—endowed a risk of experiencing a major cardiac event by a factor of more than two-fold. The finding has, not surprisingly, been strongly disputed in recent times, both on methodological grounds and because of a subsequent failure to independently replicate the results of the WCGS. Nonetheless, it demonstrated for the first time that the heart/brain nexus was amenable to systematic epidemiological investigation, and that the nexus was more than simply observationally based conjecture.

But history takes us just so far in understanding the heart/brain interface. Empirical evidence—such as that which we first saw in the WCGS—as we now know, must completely replace clinical observation alone, and over the past 45 years, this evidence has appeared in abundance. It has been derived from studies utilizing a broad array of research settings and foci, spanning the basic bioscience and psychological laboratories, the working medical facilities of hospitals and clinics, and into the territory of the general population itself. It has drawn on a plethora of methodologies and approaches, covering the fundamental laboratory-based investigations of the biomedical sciences, the experimental paradigms of psychophysiology, the invasive techniques of interventional cardiology, the use of tightly controlled clinical trials, and the rigorous field work of prospective epidemiological studies. And importantly, it has been truly multi-disciplinary in nature, uniting the specific knowledge and skills of biological scientists working in fundamental biomedicine, cardiologists, psychiatrists and psychologists, epidemiologists and statisticians, each contributing uniquely but synergistically to a common purpose—to understand the heart/brain nexus and to translate this understanding into the better clinical management of those with, or at risk of, cardiac disease.

Historically, we therefore came to ask: is psycho-social stress reliably implicated in the risk or clinical incidence of cardiac disease? Recent reviews of empirical studies now allow some solid conclusions to be drawn. Dimsdale (15), reviewing evidence on the roles of both acute and chronic stressors in relation to CVD noted three clear conclusions: first, there is overwhelming evidence that stress negatively effects the heart at an acute level; second, the evidence is strongest for stressors as triggers of coronary events; and third, the evidence on stress as a causal factor in cardiovascular disease is less strong. Steptoe and Kivimaki (16) some years later, following a careful meta-analysis of the relative strengths of the stress/heart link, provide more conclusive findings linking workplace stress to both CVD risk and clinical incidence of cardiac events. This review highlighted the cardio-pathological effects of loneliness and social isolation, and noted the roles of both anger and depression as triggers of cardiac events. And in a very recent review specifically focusing on the neurobiological causal mechanisms linking stress to CVD, Osborne et al. (17) concluded that the evidence to date now warrants the clinical use of stress reduction approaches in the mitigation of CVD risk. Each of these reviews concedes deficiencies both in the definitions and measurement of stress. Moreover, the definitive prospective study of stress and CVD in a completely unselected general population has yet to be done. Nonetheless, we have come far in the last five decades from a situation of hopeful speculation regarding stress and the heart, to a point where the systematic empirical evidence linking the two can no longer be discounted.

More recently however, attention appears to have turned to examining the more specifically experienced negative emotions, and principally depression, anxiety, and anger, usually when evident to a pathological degree, as possible causal precursors to CVD. Contemporary reviews of the evidence suggest that this was a useful move away from the more broad-brush focus on stress. Depression is clearly concomitant with clinical cardiac events for many patients. Moreover, depression is known to predict poorer outcomes in patients after diagnosed cardiac events (18). However, recent meta-analytic reviews of methodologically strong prospective studies of CVD risk clearly link major depression prospectively with both elevated risk of CVD and elevated mortality levels in those with existing incident CVD (19). This has been recently confirmed in a large (>145,000 participants) multi-center study of depression and both CVD morbidity and mortality over a 9-year period (20). It can be sensibly concluded therefore that depression, whether as a diagnosed state or as a collection of symptoms, endows some individuals with an elevated risk of both CVD morbidity and mortality. The strength of this evidence recently led the National Heart Foundation of Australia to take the position that depression must now figure among the more historically recognized risk markers for CVD (21).

The evidence is a little more equivocal in regard to anxiety and CVD, particularly in drawing causal conclusions (22, 23), but the current evidence does not discount the link. Anxiety, like depression, is clearly concomitant with clinical cardiac events, and appears related both to onset and prognosis (24). However, reviews by both Batelaan et al. (25) and Reinar et al. (26) indicated the possibility of a prospective relationship between anxiety states and an elevation in CVD incidence over time. And arguably the most persuasive evidence on anxiety and CVD comes from studies on Post-Traumatic Stress Disorder (PTSD) and CVD—the accumulation of evidence here now establishes a compelling case for that causal link (27). Perhaps the weakest evidence thus far is for the link between anger and CVD, though it is still sufficiently interesting to remain in contention. Contemporary work supports a prospective link between CVD and destructive anger (28), anger in response to stress (29), and anger proneness (30). Nonetheless, the most persuasive view of anger presently is that of a trigger to, rather than a cause of incident CVD (31, 32).

But perhaps the most contentious issue in this emerging domain of evidence addresses the postulated links between serious mental illness (psychosis) and CVD. Symptoms of psychosis—including Schizophrenia and Bipolar Disorder—have been contemporaneously linked with CVD risk or incidence (33–35). However, issues of causality remain unresolved, and it has been proposed that if such a link exists, it may be mediated through the coexistence of unhealthy or risky lifestyle factors such as cigarette smoking, inadequate nutrition and physical neglect, or even through the lipid-elevating side effects of some anti-psychotic medications (35). The possible link between psychosis and CVD risk is, therefore, yet to be fully explored.

Evidence is also now accumulating on the role of childhood adversities in the development of future health risk. This perspective, integrating findings from different disciplines, links mental disorders with CVD, and paves the way for a lifespan perspective in psychocardiology. Multiple stressors during child development affect the developed adult, thereby partly structuring their phenotypes. Adverse childhood experiences are now increasingly recognized as factors modulating health and illness across the life-course. In a landmark study (36) childhood adversities were found to increase the risk of mental disorders, CVD, and unhealthy lifestyles such as smoking, eating behaviors and low physical activity.

One theory aiming to explain the long-term sequelae of chronic stressors in childhood is the concept of *allostasis* (37). Allostasis describes the physiological responses to stressors evolved to maximize the probability of survival while limiting somatic damage. Unfortunately, such beneficial defensive responses come at a cost and, over time, repeated allostatic activity, particularly activation of stress hormone systems (38), leads to systemic somatic damage and loss of resilience to additional stressors. This damage accumulates and is known as allostatic load (39).

The notions of childhood adversities and allostasis, therefore, may stimulate a whole new theoretical direction of research, with clinical implications for both diagnosis (assessment of childhood adversities and comorbid mental disorders in patients with CVD), treatment and prevention strategies.

In this broad context then, the Special Issue of *Frontiers in Psychiatry, Psychocardiology: Exploring the Brain-Heart Interface* was conceived—and 26 original and peer reviewed papers in the area are now presented. Authors were not constrained by any pre-ordained structure—their brief was simply to report on contemporary evidence, either as original research or as critical reviews, bearing on this interface. Nonetheless, examination of the accepted submissions indicates that they aggregate under several albeit loosely bordered categories. *Early developmental psychosocial precursors* typically childhood trauma, and emotional and physical abuse (but interestingly, less so sexual abuse) were identified in relation to thromboembolic pulmonary hypertension (Lepsy et al.), pulmonary arterial hypertension (Park et al.) and congenital heart disease (Proskynitopoulos et al.) manifested later in adulthood. The over-representation of *psychological disorders (typically as depression and anxiety disorders) and stress*, in patients with thromboembolic pulmonary hypertension (Dering et al.) and those experiencing sudden cardiac arrest (Batelaan et al.) are also reported. And dysfunctional meta-cognitions more broadly are linked to pulmonary arterial hypertension (Caldarone et al.). Though causal inferences cannot be drawn for these data. Attention is also drawn, beyond the recognized effects of anxiety and depression, to the phenomenon of more broadly characterized cardiac distress in relation to longer-term recovery after a cardiac event (Jackson et al.). And we are also reminded that cognitive dysfunction too may follow cardiac events requiring surgical intervention (Vu and Smith).

Possible *psycho-biological mechanisms* also figure in the papers presented here. The central role of brain monoamines in those with both anxiety and depression has now been conclusively linked to CVD (Esler et al.). Elevated deposits of visceral adipose tissue have also been linked to CVD, possibly *via* inflammatory processes (Stapel et al.). And consistent with already published evidence, increased stress reactivity to psychosocial stimuli in the laboratory has been associated with primary hypertension (Balint et al.). This challenge-based methodology did not, however, generalize to Yohimbine induced heart rate variability in unmedicated depressed patients (Deuter et al.). The impact of CVD on both employment and Quality of Life (QoL) has been documented in patients with pulmonary hypertension (Fuge et al.; Olsson et al.) and survived myocardial infarction (Burnos and Wrzosek), and decreased QoL has also been associated with heart failure (Zormpas et al.; Anthony et al.).

The *COVID-19 pandemic* has also stimulated recent research into CVD. Papers alerting us to the impact of the pandemic on the availability of medical services to patients with congenital heart disease (Akkermann et al.), and the negative consequences of COVID lockdown on physical activity and sleep patterns of children (as potential future CVD risk; Olive et al.) raise areas of future research. The pandemic did not, however, appear to negatively impact on mental distress or QoL in patients with Pulmonary Arterial Hypertension (Park et al.).

And finally, it was pleasing to see that *interventions for mental illness* in patients with cardiac conditions featured prominently in contributions to this Special Issue. Interventions using psychotropic medications remain important though, in the light of well-demonstrated cardio-toxicity in some agents, they must be used with considerable caution (Kahl et al.). There is also a raft of (relatively) cardio-specific issues to do with adherence to medication which must be recognized (Halling et al.). Psychotherapeutic interventions for both anxiety and depression in patients with CVD are, however, now becoming increasingly employed, and both Meta-cognitive Therapy (Wells et al.; Gebhardt et al.; Caldarone et al.) and Transdiagnostic CBT (Tully et al.) show significant promise as effective psycho-cardiological interventions.

In this light, we believe that our Special Issue gives rise to inspiring new ideas concerning all aspects of psychocardiology. Screening for mental disorders, disease associated distress, and childhood trauma is recommended in the workup of patients. Psychotherapeutic interventions are recommended at all levels of cardiovascular treatment, and in severe cases, psychopharmacological drug treatment can carefully be considered. However, more research is clearly merited to ensure fully convincing evidence able to be integrated into current treatment guidelines.

## Author contributions

DB, KGK, and MA made substantial contributions to the conception and idea of the Editorial, drafted the work and revised it critically for important intellectual content, provided approval to the final version, and agreed to be accountable for all aspects of the work.

## Conflict of interest

The authors declare that the research was conducted in the absence of any commercial or financial relationships that could be construed as a potential conflict of interest.

## Publisher's note

All claims expressed in this article are solely those of the authors and do not necessarily represent those of their affiliated organizations, or those of the publisher, the editors and the reviewers. Any product that may be evaluated in this article, or claim that may be made by its manufacturer, is not guaranteed or endorsed by the publisher.
